# Binary and Multi-Class Classification of Colorectal Polyps Using CRP-ViT: A Comparative Study Between CNNs and QNNs

**DOI:** 10.3390/life15071124

**Published:** 2025-07-17

**Authors:** Jothiraj Selvaraj, Fadhiyah Almutairi, Shabnam M. Aslam, Snekhalatha Umapathy

**Affiliations:** 1 Department of Biomedical Engineering, College of Engineering and Technology, SRM Institute of Science and Technology, Kattankulathur, Chengalpattu 603203, India; js0740@srmist.edu.in; 2 Department of Information Systems, College of Computer and Information Sciences, Majmaah University, Al Majmaah 11952, Saudi Arabia; fma.almutairi@mu.edu.sa; 3 Department of Information Technology, College of Computer and Information Sciences, Majmaah University, Al Majmaah 11952, Saudi Arabia

**Keywords:** colorectal polyp classification, CRP-ViT, vision transformer, CNN, QNN, colonoscopy images, quantum computing

## Abstract

Background: Colorectal cancer (CRC) is a major contributor to cancer mortality on a global scale, with polyps being critical precursors. The accurate classification of colorectal polyps (CRPs) from colonoscopy images is essential for the timely diagnosis and treatment of CRC. Method: This research proposes a novel hybrid model, CRP-ViT, integrating ResNet50 with Vision Transformers (ViTs) to enhance feature extraction and improve classification performance. This study conducted a comprehensive comparison of the CRP-ViT model against traditional convolutional neural networks (CNNs) and emerging quantum neural networks (QNNs). Experiments were conducted for binary classification to predict the presence of polyps and multi-classification to predict specific polyp types (hyperplastic, adenomatous, and serrated). Results: The results demonstrate that CRP_QNN_-ViT achieved superior classification performance while maintaining computational efficiency. CRP_QNN_-ViT achieved an accuracy of 98.18% for training and 97.73% for validation on binary classification and 98.13% during training and 97.92% for validation on multi-classification tasks. In addition to the key metrics, computational parameters were compared, where CRP_QNN_-ViT excelled in computational time. Conclusions: This comparative analysis reveals the potential of integrating quantum computing into medical image analysis and underscores the effectiveness of transformer-based architectures for CRP classification.

## 1. Introduction

Colorectal cancer (CRC) ranks among the prevalent and fatal cancers across the globe, with its early stages often marked by the presence of colorectal polyps [1,2]. The timely detection and classification of these polyps during colonoscopy examinations are crucial for preventing malignant transformation [2,3,4]. However, the manual interpretation of colonoscopy images can be subjective, labor-intensive, and susceptible to differences in interpretation among observers [5,6]. As a result, the integration of Artificial Intelligence (AI) into clinical diagnostics has gained increasing attention for improving the accurate detection of anomalies [7,8,9,10].

Convolutional neural networks (CNNs) have traditionally been the backbone of image classification tasks, including medical imaging [11,12]. Their hierarchical feature extraction capabilities have enabled them to detect fine-grained patterns in endoscopic images [13]. Also, Vision Transformers (ViTs) have gained prominence as an effective alternative, utilizing self-attention mechanisms to model long-range interactions and spatial relationships within an image [14,15].

In this study, we propose a novel hybrid architecture, CRP-ViT, that combines the strengths of convolutional layers for local feature learning with the global attention capabilities of Vision Transformers. This unified model is designed to enhance the classification of CRP from colonoscopy images. In addition to this, we explore the emerging paradigm of quantum neural networks (QNNs), which utilize principles of quantum computation to enable complex pattern recognition and data encoding within quantum states. While still in their infancy, QNNs offer promising advantages in terms of computational parallelism and robustness in high-dimensional feature spaces.

### 1.1. CNN

**Figure 1:**
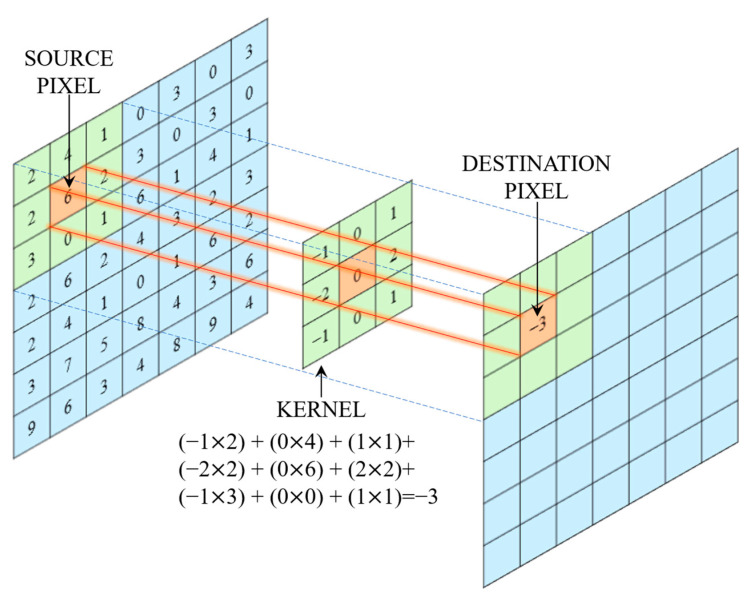
Illustration of a 2D convolution operation in a CNN using a 3 × 3 kernel.

CNNs represent a specialized architecture within the broader domain of DL, particularly tailored for image analysis and computer vision tasks [16]. Inspired by the structure and functioning of the human visual cortex, CNNs are adept at recognizing and interpreting spatial hierarchies and patterns within images [17]. Their architecture is composed of several interconnected layers, each performing a specific function that contributes to the model’s ability to learn from image inputs [12,18]. The core components of a CNN includes convolutional layers, activation functions, pooling layers, fully connected layers, back propagation, and optimization [19]. The fundamental building block of a CNN is the convolutional layer, which employs a series of learnable filters or kernels that convolve across the input image to detect local features such as edges, textures, corners, and more complex shapes [12]. The core operation of a 2D convolution layer in a CNN is provided in Figure 1.

The input is a two-dimensional grid representing an image or feature map upon which a sliding window, defined by a kernel or filter, moves across the input data, performing element-wise multiplication followed by summation at each location [12]. Each filter is sensitive to a particular kind of visual pattern, and multiple filters allow the network to learn a diverse set of features at various levels of abstraction [20]. Following the convolution, activation functions, typically non-linear functions such as the Rectified Linear Unit (ReLU), are applied to introduce non-linearity into the model, which is crucial for learning intricate patterns and relationships in the data [21]. To manage the high dimensionality of feature maps and to make the network more computationally efficient, pooling layers are introduced [22]. These layers perform a downsampling operation, reducing the spatial dimensions of the feature maps while preserving the most important information [23]. After successive stages of convolution, activation, and pooling, the resulting features are flattened into a one-dimensional vector and fed into one or more fully connected layers (dense layers) [19,24]. These layers function as the decision-making component of the network, integrating the learned spatial features to perform classification tasks [25]. The learning process in CNNs involves forward propagation, where inputs pass through the network to produce an output, and back propagation, a mechanism that adjusts the model parameters by propagating the error gradients backward through the network [26]. This optimization process is typically governed by algorithms such as Adam, which iteratively minimize the loss function to improve prediction accuracy [27,28].

### 1.2. QNN

**Figure 2:**
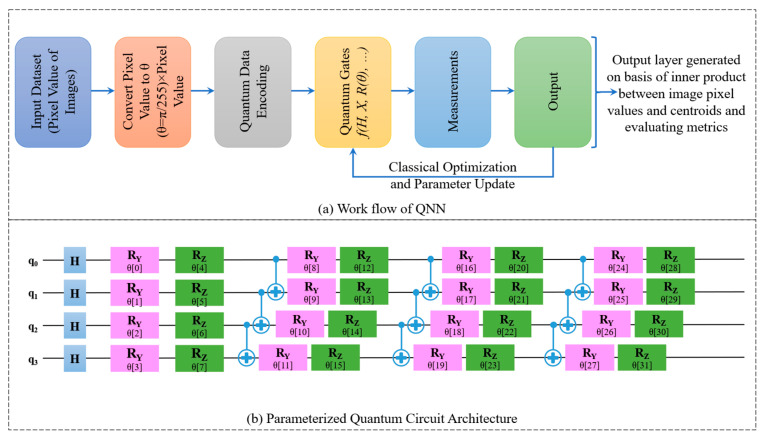
Quantum circuit for DL: (**a**) Work flow (**b**). Parameterized quantum circuit architecture.

Recent advancements in quantum computing have paved new pathways in the field of AI, particularly in the development of quantum neural networks (QNNs) [29]. Unlike classical neural networks, QNNs harness quantum mechanical phenomena—namely superposition, entanglement, and quantum interference—for processing information in a high-dimensional Hilbert space, thereby enabling more complex pattern recognition capabilities with fewer resources [30]. The quantum workflow is provided in Figure 2a encompassing Quantum Data Encoding, where the pixel intensities of the input image are transformed into quantum states through angle encoding schemes allowing quantum systems to represent large amounts of information compactly [31,32]. Instead of traditional neurons and weights, QNNs use quantum gates organized in quantum circuit layers to transform quantum states [33]. These transformations create entangled and superposed states that enable parallel computation across multiple possibilities [34]. After quantum operations, measurements are taken to collapse the quantum state into classical outcomes, which are interpreted for tasks like classification [35]. Due to the current limitations of quantum hardware, many QNNs are implemented as hybrid models [36]. A quantum circuit acts as a layer within a classical neural network and optimization is performed using classical techniques, guided by measurement outcomes from the quantum layer [37]. The Quanvolutional layers used in this study were implemented using PennyLane with Qiskit, a quantum simulator backend, which executes real quantum circuit operations on classical hardware. The design adheres to valid quantum logic gate principles, simulating actual qubit behavior within the constraints of current simulation environments.

In this study, the 4-qubit QNN architecture was utilized for the classification of polyps in colonoscopy images, as shown in Figure 2b. Each qubit in the network represents a quantum bit capable of existing in a superposition of states, which exponentially increases the representational capacity of the system [38]. The adoption of a 4-qubit design balances computational complexity and expressiveness, rendering it compatible for present Noisy Intermediate-Scale Quantum (NISQ) devices while maintaining scalability for future hardware improvements [39]. The input medical images are first preprocessed using classical techniques. Subsequently, the angle encoding is used to map classical features *x*_i_ ∈ [0, 1] into quantum states using rotation gates. Specifically, each feature is embedded into a qubit using the parameterized gates R_y_(π*x*_i_) and R_z_(π*x*_i_) [40]. The complete normalized feature vector is represented as per Equation (1).

(1) $$x=[x_{1},\;x_{2},\ldots ,x_{n}]$$

where *x_i_* denotes the *i*th normalized scalar feature, where *x_i_* ∈ [0, 1] and n indicates the number of features.

Each *x_i_* is encoded into a quantum state using parameterized rotation gates applied to qubits initialized in the ground state ∣0⟩. The Y-axis and Z-axis rotation gates R_y_(π*x*_i_) and R_z_(π*x*_i_) are represented by Equations (2) and (3), respectively.

(2) $$R_{y}\left(\pi x_{i}\right)=\left[\begin{array}{cc} cos(\theta_{i}/2) & -sin(\theta_{i}/2)\\ sin(\theta_{i}/2) & cos(\theta_{i}/2) \end{array}\right]$$

(3) $$R_{z}\left(\pi x_{i}\right)=\left[\begin{array}{cc} e^{-i\theta_{i}/2} & 0\\ 0 & e^{i\theta_{i}/2} \end{array}\right]$$

For multiple features, a system of n qubits is used. The full composite quantum state of the system is given by the tensor product as provided in Equation (4).

(4) $$\left|\Psi \rangle ={⨂}_{i=1}^{n}\;R_{y}\left(\pi x_{i}\right)R_{z}\left(\pi x_{i}\right)|0\right\rangle$$

The symbol ⨂ represents the tensor (Kronecker) product operator, combining individual qubit states into a multi-qubit system.

Following the encoding, CNOT entanglement operations are applied to capture complex inter-feature dependencies. The QNN is trained using a variational quantum circuit (VQC) paradigm, where the parameters of the rotation gates are iteratively updated using classical optimization algorithms [41]. The output from quantum measurements is used to calculate class probabilities for multi-class classification.

In the present investigation, a shallow quantum circuit was utilized due to noise sensitivity and computational complexity in simulating quantum operations. Specifically, each 4-qubit circuit consisted of rotation gates for encoding followed by entanglement via CNOT gates. Deeper circuits were avoided to maintain stability and computational feasibility on classical simulators, acknowledging the exponential growth in resources required as the qubit count or depth increases.

### 1.3. Survey of Literature

The integration of AI in gastrointestinal endoscopy has significantly enhanced the detection and classification of CRPs, a key step in the prevention of CRC [42]. Over the past decade, various DL models have been deployed to improve the diagnostic accuracy of colonoscopy procedures, with CNNs emerging as the dominant approach due to their high performance in image classification tasks [43,44].

Several studies have demonstrated the utility of CNNs in CRP classification. For instance, Urban et al. [45] developed a real-time DL system that achieved expert-level performance in detecting polyps in colonoscopy videos. Adrian Krenzer et al. [46] developed an automated method to classify polyps according to the NICE and Paris classifications. Similarly, Balasubramani et al. [47] trained a CNN model on the Kvasir and CVC-ClinicDB datasets, achieving high precision and recall rates in distinguishing adenomatous from non-adenomatous polyps. Sena Busra Yengec-Tasdemir et al. [48] attempted to classify polyp categories based on histopathological images. The histopathological images required invasive procedures of biopsy to be taken. The authors achieved an accuracy of 87.1% with a custom dataset and 70.3% with the publicly available UniToPatho dataset. The authors limited their work to hyperplastic and adenoma polyps. Pradipta Sasmal et al. [49] proposed generative adversarial networks for the UniToPatho dataset and achieved an accuracy of 87.50%. The study carried out by Thomas De Carvalho et al. [50] focused on the NICE classification, for categorizing polyps as either hyperplastic or adenomas or cancer. Trained with multiple datasets, the classifier produced 92% accuracy on a custom dataset and 88% on the Piccolo dataset. Despite their success, CNNs are inherently limited by their confined receptive fields, which may hinder their ability to capture global contextual information crucial for complex image structures like polyps with subtle variations.

To overcome these limitations, Vision Transformers (ViTs) have been introduced as a viable alternative. Dosovitskiy et al. [51] pioneered the use of transformer-based models for image recognition by applying the self-attention mechanism to image patches. Haq et al. [52] applied ViT architectures for gastrointestinal disease detection, reporting improvements in classification accuracy over traditional CNNs. However, transformers generally demand extensive training datasets and significant computational power, which may limit their application in resource-constrained clinical settings.

Parallel to these advancements, quantum neural networks (QNNs) have emerged as a novel computational paradigm that combines quantum mechanics and neural networks [53]. Leveraging quantum bits (qubits) and quantum gates, QNNs have the potential to encode complex feature spaces with fewer resources, enabling exponential speed-ups for certain tasks [54]. Schuld and Killoran [55] proposed a hybrid quantum–classical model that successfully performed classification tasks on medical datasets. Ovalle-Magallanes et al. [40] introduced a learnable quantum angle encoding framework for the classification of ECG and EEG signals, which are critical for neurological and cardiac imaging diagnostics. While not limited to imaging alone, Meghanath et al. [32] proposed a quantum deep CNN for real-time, high-dimensional visual data analysis in safety-critical systems; their approach has potential translational relevance for diagnostic radiology, where real-time and accurate visual pattern recognition is essential. Awujoola et al. [53] applied QNNs to the early detection of breast and lung cancer by classifying histopathological and cytology images, showcasing improved sensitivity in differentiating malignant from benign tissues. More recently, QNNs have been explored for medical image analysis, such as in cancer detection and genomics, although most studies remain at a proof-of-concept level due to current limitations in quantum hardware.

Despite the promise shown by individual models, very few studies have undertaken a comparative evaluation of CNNs, ViT-based models, and QNNs for CRP classification. Furthermore, hybrid models combining CNNs and ViTs remain underexplored in the gastrointestinal imaging domain. This research aims to bridge this gap by proposing a CRP-ViT hybrid model and evaluating its performance against both classical CNNs and emerging QNNs, thereby offering a comprehensive perspective on the strengths and limitations of each approach in CRP classification.

### 1.4. Problem Identified from the Literature

Most existing studies on polyp detection from colonoscopy images primarily rely on classical deep learning architectures. In contrast, this work explores the application of quantum computing to enhance both computational efficiency and detection performance. Furthermore, the majority of prior research focuses on binary classification, aiming only to identify the presence or absence of polyps, rather than distinguishing between specific polyp types. Even in studies that address multi-class classification, the problem is often decomposed into a series of binary tasks. Additionally, these multi-class approaches typically categorize polyps based on superficial attributes such as color and texture, without providing insight into the clinical severity or pathological significance of the lesions.

### 1.5. Aim and Objective of This Article

The aim of this study is to conduct a comparative evaluation between two architectures: CRP-ViT integrated with a classical CNN and its quantum-enhanced counterpart employing a QNN. The models were assessed on independent image datasets beyond those used during training and validation to examine their generalization capabilities. The objective of this research is to highlight the potential advantages of quantum-assisted learning over conventional CNN- and transformer-based methods. Building upon the previously published CRP_CNN_-ViT framework, the authors introduce a modified architecture CRP_QNN_-ViT by replacing the convolutional layers with Quanvolutional layers. The goal was to train and validate this network for both binary and multi-class classification tasks and benchmark its performance against results reported in the base reference study. The findings are intended to contribute to the advancement of more accurate and generalizable diagnostic tools for CRC screening.

### 1.6. Contribution of This Article

The contributions of the authors include the following:

- A diverse CRP image dataset is aggregated by combining publicly available sources with self-curated real-time clinical colonoscopy images to enhance the dataset’s variability in training and evaluating DL models in the detection of CRP.
- A novel architectural modification is proposed by replacing classical convolutional layers in the original CRP_CNN_-ViT model with Quanvolutional layers, resulting in a hybrid quantum model named CRP_QNN_-ViT. This integration aims to deploy quantum computational advantages such as parallelism and entanglement for improved feature representation and classification performance.
- Both the classical (CRP_CNN_-ViT) and quantum (CRP_QNN_-ViT) models are employed for binary (polyp or normal) and multi-class classification (hyperplastic or adenoma or serrated or normal).
- A detailed comparative analysis is conducted between the CRP_CNN_-ViT and CRP_QNN_-ViT models. Performance is evaluated using standard classification metric, as well as computational complexity parameters to highlight the trade-offs between classical and quantum-enhanced models in practical deployment scenarios.
- An ablation study is performed on the CRP_QNN_-ViT model to systematically evaluate the impact of quantum components on CRP-ViT architectures.

### 1.7. Outline of This Article

This article is organized to systematically present the development and evaluation of a hybrid classical–quantum model for CRP classification. Section 1 is the Introduction, providing foundational insights into CNNs and QNNs, followed by a comprehensive literature survey, the identification of existing research gaps, and a clear statement of the study’s aims, objectives, and contributions. Section 2 provides the Materials and Methods, detailing the methodology in two phases including binary and multi-class classification, followed by the design of the proposed CRP_QNN_-ViT architecture, performance evaluation strategies including cross-validation, and a comparative analysis between the CRP_CNN_-ViT and CRP_QNN_-ViT models. The quantitative findings from both classification phases are presented in Section 3. This is followed by Section 4 in which the results are interpreted in greater depth including the current limitations and challenges faced in implementing quantum-based models. Finally, Section 5 summarizes the key findings and underscores the future potential of integrating quantum computing with deep learning for medical image analysis.

## 2. Materials and Methods

**Figure 3:**
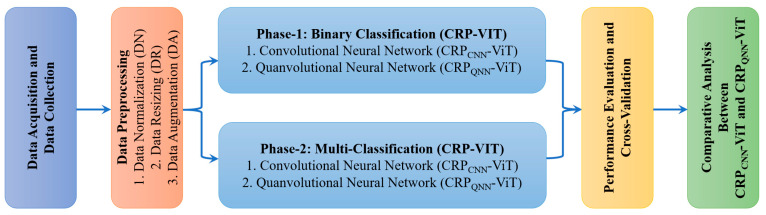
Work flow of the proposed research study.

The pipeline of this research is divided into two phases, each contributing to the comparative analysis between classical and quantum deep learning models, as illustrated in Figure 3. The initial stages in both the phases involves the collection of datasets that are representative of the CRP and preprocessing. Phase 1 focuses on the binary classification of CRP (polyp vs. non-polyp), while phase 2 addresses the multi-class classification of polyp subtypes. Both phases adopt the CRP-ViT framework, where two distinct DL paradigms, including CNNs and QNNs, are explored.

In the previous works carried out by the authors, CRP-ViT integrated with a CNN backbone was developed to address both binary [56] and multi-class [57] classification tasks in CRP analysis involving identifying the presence or absence of polyps and distinguishing between polyp types, respectively. Building upon this foundational work, the current study introduces an enhanced version of the CRP_QNN_-ViT model which incorporates a Quanvolutional neural network (QNN) as its feature extraction backbone. The QNN-based approach leverages quantum-inspired convolutional operations to potentially capture more intricate patterns and relationships within medical image data, offering an innovative alternative to traditional CNN architectures. To ensure an equitable performance comparison between the proposed CRP_QNN_-ViT and the previously developed CRP_CNN_-ViT models, the experimental design adheres to the same protocols, datasets, preprocessing steps, and evaluation strategies established in the earlier study.

All deep learning (DL) experiments, including model training and evaluation, were implemented and executed on the Google Colaboratory (Colab) platform. The entire implementation was carried out using the Python (V3.10) programming language, utilizing various open-source libraries and frameworks optimized for DL workflows.

This section outlines the dataset used for experimentation, preprocessing techniques, the architecture of the proposed CRP-ViT model, and the baseline CNN and QNN architectures used for comparative analysis. It also defines the evaluation metrics employed to measure model performance.

### 2.1. Phase 1: Binary Classification

**Figure 4:**
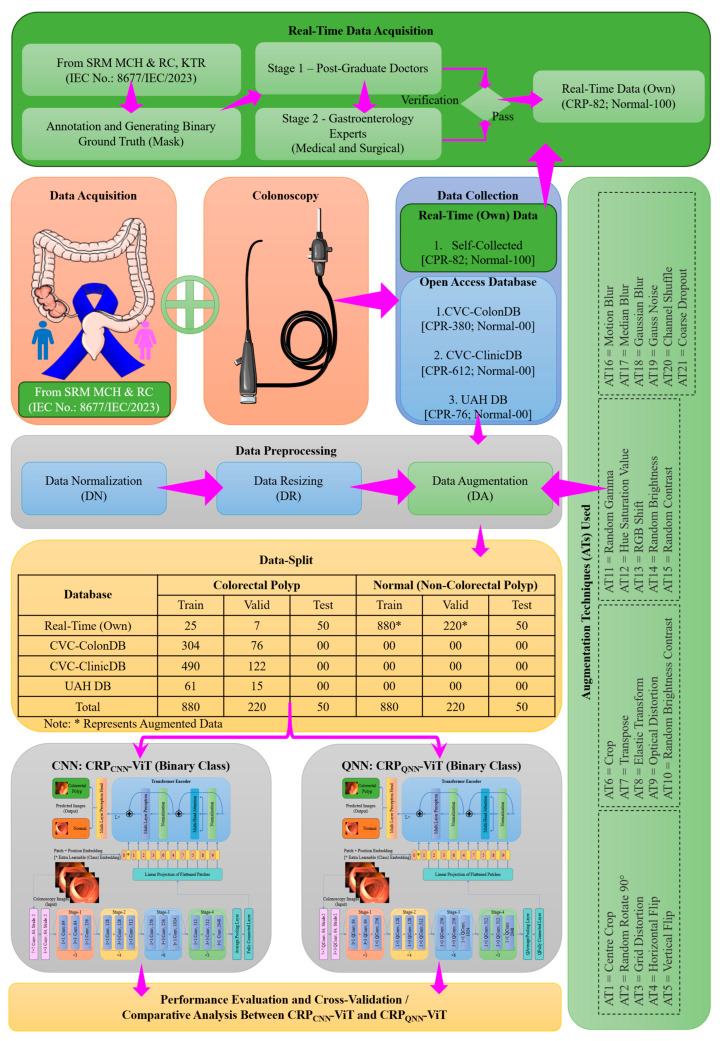
Work flow of phase 1: binary classification.

The workflow of the phase 1 involving binary classification is detailed in Figure 4. The dataset employed in this study integrates four distinct sources to ensure comprehensive and diverse representation for CRP analysis. The first source is a real-time clinical database developed through collaboration with SRM Medical College Hospital & Research Centre (SRMC & RC), comprising a total of 182 annotated colonoscopy images (82 polyp and 100 normal), obtained under institutional ethical approval (IEC No.: 8677/IEC/2023). The manual annotation of clinical images is inherently subjective, often leading to inter-observer variability, even among experts. While expert validation enhances the quality of annotations, it cannot entirely eliminate the bias introduced by either individual interpretation styles or clinical experience. Such variability can result in noisy labels, which may impact the consistency of the training data and ultimately affect the generalization ability of DL models. To mitigate these issues, our study employed a two-stage annotation strategy. In Stage 1, two medical practitioners with four years of postgraduate experience in gastroenterology annotated the data. In Stage 2, two senior professors with a combined decade of experience in medical education and clinical practice reviewed and verified the annotations. All annotators had access to corresponding pathology and biopsy reports to support their decisions. To reduce subjectivity and enhance label reliability, experts in each stage collaboratively reviewed each image and any disagreements were resolved through discussion and consensus. The finalized dataset comprised 82 polyp images and 100 normal images, serving as the real-time dataset (DS-1) [56].

In addition to this real-time dataset, three publicly available benchmark datasets were utilized: CVC-ColonDB [58], CVC-ClinicDB [59], and the UAH DB [60]. The CVC-ColonDB includes 380 annotated frames extracted from colonoscopy videos of 13 patients, providing detailed pixel-wise polyp annotations. The CVC-ClinicDB contributes a larger dataset of 612 images sourced from 23 patients, offering a broader and more diverse sample of polyp appearances across various anatomical regions. The UAH DB comprises 76 high-resolution colonoscopy images, each clinically annotated and categorized into specific histological types: hyperplastic, adenomatous, and sessile serrated polyps. However, since phase 1 of this study was dedicated to binary classification—distinguishing between polyp and non-polyp (normal) classes—all histological subtypes in the UAH DB were unified and treated collectively under the single polyp category.

To ensure consistency and to standardize the spatial dimension of the input images provided to CRP-ViT, all the colonoscopy images considered in this study were uniformly resized. Subsequently, each resized image was normalized to scale pixel intensity values between 0 and 1 [4]. Notably, 100 images (50 polyp, 50 normal) out of 182 real-time datasets were reserved as an unseen test set for model evaluation. The dataset, except the images reserved for testing, was split into 80:20 and used for training and validation, respectively. All the open-source datasets contained only polyp images; hence, normal-class images were supplemented via augmentation techniques to balance the training and validation setup. The normal images of the real-time dataset were augmented using 21 techniques to produce 1100 normal images in total. CRP_CNN_-ViT and CRP_QNN_-ViT were validated with performance metric assessment.

### 2.2. Phase 2: Multi-Classification

**Figure 5:**
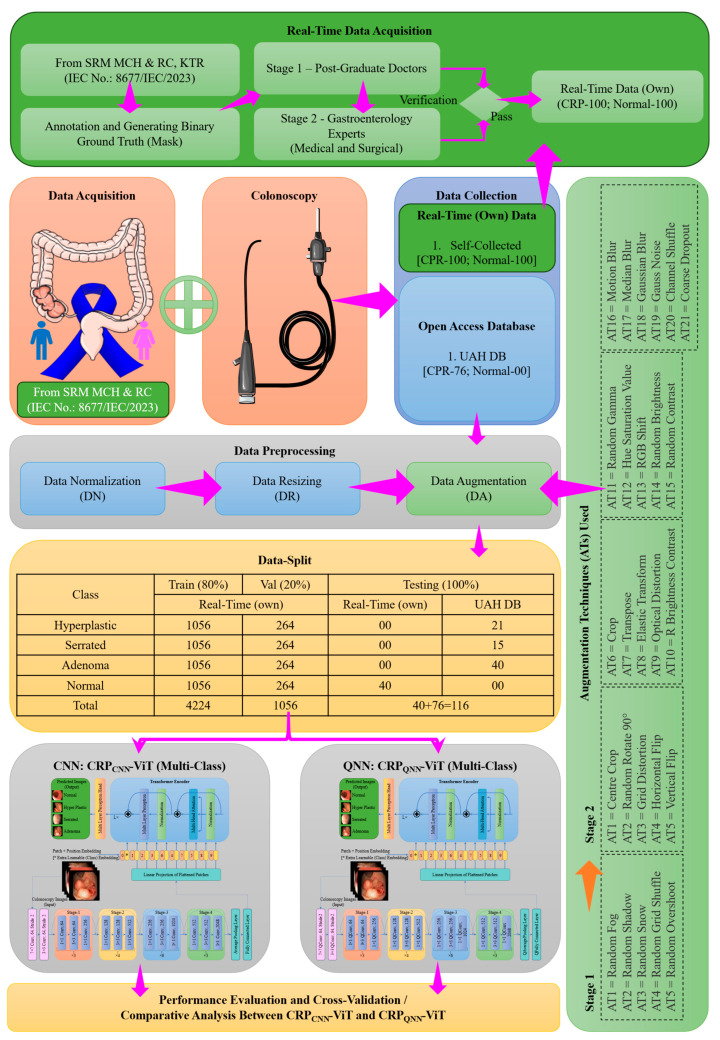
Work flow of phase 2: multi-classification.

In phase 2, a multi-classification task was undertaken, targeting four categories: hyperplastic, adenomatous, serrated, and normal. It is crucial to emphasize that the datasets used in phase 1 (DS-2 and DS-3) do not include the specific classes of polyps and therefore were inapplicable for the current multi-class classification paradigm. The workflow of phase 2 encompassing multi-classification is illustrated in Figure 5. Following the same ethical clearance obtained for phase 1, additional images were acquired to expand the number of CRP images from 82 to 100. Phase 2 utilized two datasets: (1) a self-curated real-time dataset consisting of 200 colonoscopy images (100 CRP and 100 normal) and (2) a publicly available dataset from the UAH DB (DS-4) [60], contributing 76 CRP images. All images underwent a preprocessing pipeline, including data normalization (DN) and dimensional resizing (DR). Following preprocessing, 40 normal images of real-time dataset and UAH DB CRP images were reserved for testing. In multi-classification tasks, data imbalance poses a significant challenge by causing the model to become biased toward majority classes, thereby compromising its ability to accurately learn and classify minority-class instances. The limited representation of minority classes in training data leads to inadequate feature learning, increasing the likelihood of misclassification and reducing the model’s generalization capability. Data augmentation aids in mitigating the effects of data imbalance in multi-classification tasks by increasing the number of training samples for underrepresented classes. In the present study, a two-stage augmentation approach was employed: Stage 1 involved balancing the dataset by equalizing the number of images across all classes, while Stage 2 focused on further augmenting the training samples to enhance the model’s learning capacity and robustness. After augmentation, the dataset was partitioned with an 80:20 split for training and validation, respectively. The complete data-splitting strategy has been detailed in the authors’ previously published work [57].

### 2.3. Proposed Architecture

**Figure 6:**
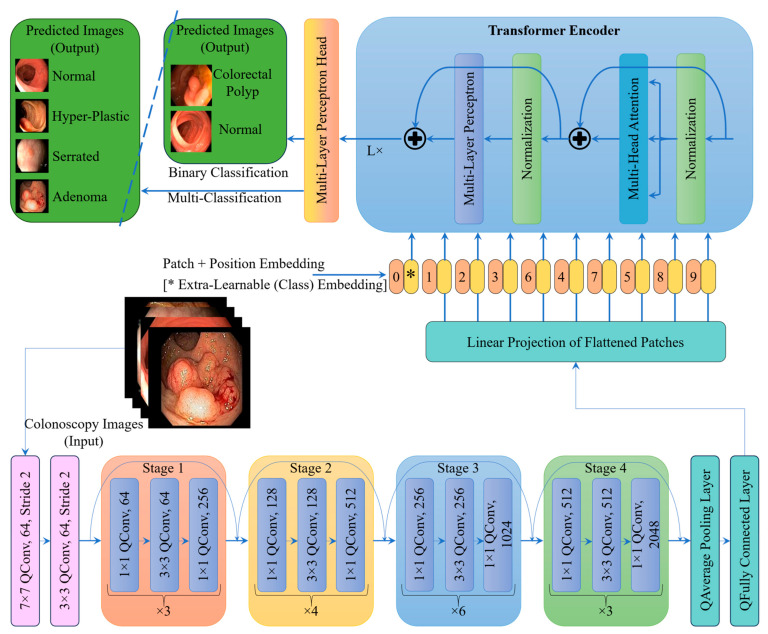
Work flow of the proposed architecture (CRP_QNN_-ViT).

Figure 6 illustrates the proposed CRP_QNN_-ViT framework designed to classify colonoscopy images into four diagnostic categories considered in this research. CRPQNN-ViT is similar to CRP_CNN_-ViT in its architecture except that Quanvolutional layers are used instead of convolutional layers. The CRP_QNN_-ViT model integrates Quanvolutional- (ResNet50) and transformer- (ViT) based components to leverage both local and global feature representations. The architecture initiates with an input stage, where colonoscopy images are processed through a series of Quanvolutional layers. The core processing component incorporates a four-stage Quanvolutional neural network (QNN) backbone, modeled on the ResNet-50 architecture and structured using bottleneck residual blocks. Each stage comprises multiple Quanvolutional layers that include combinations of 1 × 1 and 3 × 3 Quanvolutions, as detailed in Table 1. These layers are configured with progressively increasing filter depths to capture hierarchical feature representations. To efficiently manage the spatial dimensions and facilitate deeper learning, stride Quanvolutions are employed for downsampling at strategically selected stages within the network. Upon the completion of feature extraction in the QNN backbone, the resulting feature maps are flattened and converted into a sequence of fixed-size embedded patches, which serve as input to the transformer module. To preserve spatial dependencies across the image domain, positional encodings are integrated with the embedded patches. Furthermore, a learnable classification token is prepended to the sequence, enabling the ViT to aggregate global contextual information necessary for decision-making. Both the CRP_CNN_-ViT and CRP_QNN_-ViT models were trained, and their performance metrics were evaluated and compared comprehensively.

### 2.4. Performance Evaluation and Cross-Validation

**Figure 7:**
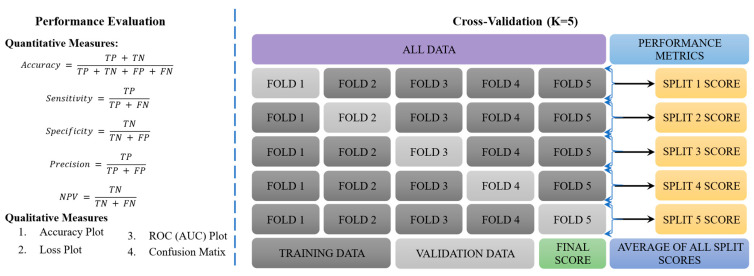
Performance evaluation and 5-fold cross-validation.

Building upon previously published research [56,57] that employed the CRP_CNN_-ViT framework, this study evaluates the performance of the proposed CRP_QNN_-ViT architecture as shown in Figure 7. The objective of this study was to compare the classification capabilities of both the CRP_CNN_-ViT and CRP_QNN_-ViT architectures in the context of CRP detection from colonoscopy images. A comprehensive comparative analysis was conducted between the CRP_QNN_-ViT and the CRP_CNN_-ViT models to assess improvements in classification accuracy in CRP detection. To ensure a robust and unbiased evaluation, a comprehensive cross-validation strategy was employed. This included partitioning the dataset into 5 folds to facilitate repeated training and validation across the data splits, thereby reducing variance and mitigating overfitting [4]. Both models, CRP_CNN_-ViT and CRP_QNN_-ViT, were trained using the same dataset splits and hyperparameter configurations to ensure a fair and controlled comparison.

### 2.5. Comparative Analysis Between CNN and QNN

In addition to the conventional performance metrics, a comprehensive comparative analysis was conducted to evaluate the architectural efficiency of the proposed CRP_CNN_-ViT and CRP_QNN_-ViT models. This assessment focused on key computational and resource-oriented parameters that significantly impact the practical deployment and scalability of DL models, particularly in real-time or resource-constrained healthcare environments. The parameters considered in this investigation included computational speed, floating-point operations per second (FLOPs), total trainable parameters, and memory usage.

## 3. Results

The experimental results reported in this study are derived from the implementation and evaluation of the proposed CRP_QNN_-ViT architecture. For a fair and consistent comparison, the performance metrics associated with the CRP_CNN_-ViT model are adopted from our previously published work [56,57].

### 3.1. Phase 1: Binary Classification

The performance achieved by the CRP_QNN_-ViT using an 80:20 split is detailed in Table 2. During the training and validation phase, the model achieved accuracies of 98.18% and 97.73%, demonstrating its capability to effectively learn discriminative features. The sensitivity and NPV were recorded at 98.64% and 98.61%, respectively, during validation, indicating a strong ability to correctly identify positive cases and minimize false negatives. The specificity reached 96.82%, while the precision was 97.88%, highlighting the model’s balanced performance in detecting both positive and negative instances.

**Figure 8:**
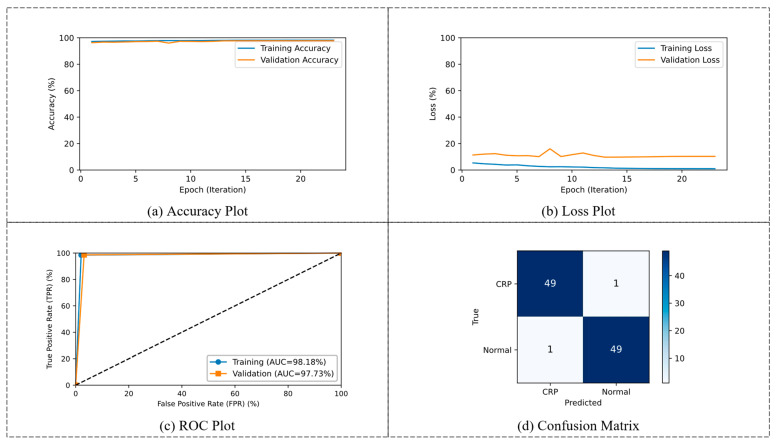
Visualization of CRP_QNN_-ViT binary classification performance. (**a**) Accuracy plot, (**b**) loss plot, (**c**) ROC plot, and (**d**) confusion matrix for test results.

The performance of the CRP_QNN_-ViT model for binary classification between CRP and normal images is illustrated in Figure 8. The training and validation accuracy curves indicate rapid convergence within 23 epochs, with both curves stabilizing above 98%, suggesting effective learning and minimal overfitting. The loss curves further confirm model stability, with the training and validation losses remaining consistently low throughout the training process. The Receiver Operating Characteristic (ROC) analysis demonstrates the model’s strong discriminative ability, with AUC values of 98.18% for training and 97.73% for validation. Additionally, the confusion matrix of test images highlights the classification performance, with 49 out of 50 CRP and 49 out of 50 normal samples correctly identified, resulting in a testing classification accuracy of 98%.

The classification performance across the five folds is summarized in Table 3. Almost Similar trends of performance were observed among all the folds of the 5-fold cross-validation. The mean and standard deviation of the performance metrics across the folds indicate that the CRP_QNN_-ViT model maintained stable performance across different data partitions.

The difference in performance between the 80:20 split and 5-fold cross-validation is tabulated in Table 4. The observed differences between the two approaches were minimal, while accuracy remained identical. These negligible deviations demonstrate the model’s high degree of stability across different validation techniques and reinforce its potential applicability in clinical settings for CRP classification.

### 3.2. Phase 2: Multi-Classification

The CRP_QNN_-ViT model was further assessed for its effectiveness in performing multi-class classification tasks, as presented in Table 5. Specifically, the model was employed to accurately differentiate and predict multiple categories, including various types of CRP as well as normal tissue. The class-wise performance achieved by CRP_QNN_-ViT using an 80:20 split during both the training and validation phases over 29 out of 50 epochs, under the Adam optimizer configuration, is presented. The overall accuracy during training and validation were 98.13% and 97.92%.

**Figure 9:**
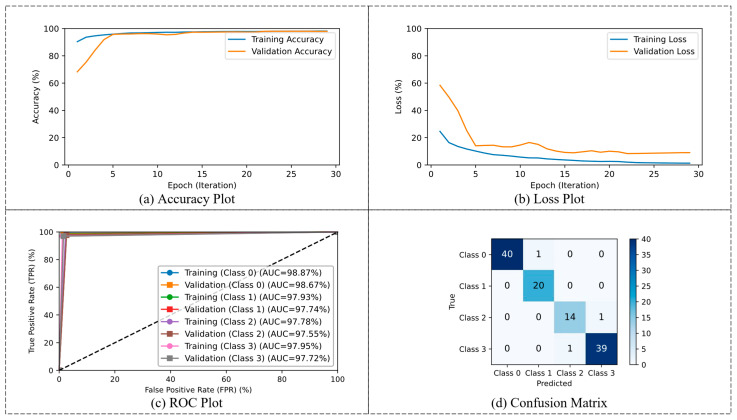
Visualization of CRP_QNN_-ViT multi-classification performance. (**a**) Accuracy plot, (**b**) loss plot, (**c**) ROC plot, and (**d**) confusion matrix for test results.

The multi-class classification performance of the CRP_QNN_-ViT model is depicted in Figure 9, showcasing its training behavior and evaluation metrics. The accuracy curves reveal that both training and validation accuracies converge quickly, indicating the model’s strong generalization capability across multiple polyp classes. The loss plot demonstrates a consistent reduction in both training and validation losses, reflecting efficient learning and model stability. The ROC curves for each class highlight high discriminative power, with AUC scores exceeding 97% across all classes. The confusion matrix of the test images further validates model performance, showing high classification accuracy across all five classes, with minimal misclassifications.

The class-wise performance achieved by CRP_QNN_-ViT during 5-fold cross-validation is provided in Table 6. Across the five folds (K = 1 to K = 5), the CRP_QNN_-ViT consistently demonstrated high classification performance. The overall classification accuracy across all classes and folds was approximately 97.94 ± 0.35. The variance among the folds was minimal, indicating that CRP_QNN_-ViT maintained its performance across all the folds irrespective of the data partition. The difference in performance of CRP_QNN_-ViT using an 80:20 split and 5-fold cross-validation is provided in Table 7. The observed consistency in the performance of CRP_QNN_-ViT using an 80:20 split and K-fold cross-validation again confirms the model’s strong generalization capability.

## 4. Discussion

### 4.1. Phase 1: Binary Classification

Table 8 provides a comparative evaluation of the computational complexity and execution time for the CRP_CNN_-ViT and CRP_QNN_-ViT models considered for binary classification. The CRP_QNN_-ViT model exhibits a more lightweight architecture, with a total of 86 million parameters, which are significantly fewer than the 132 million parameters required by CRP_CNN_-ViT. This reduction is reflected in computational demands, as CRP_QNN_-ViT performs only 8.5 Giga Multiply–Accumulate Operations (MACs) and 17 Giga floating-point operations (FLOPs), compared to 11.6 Giga MACs and 23.2 Giga FLOPs for the classical model. Furthermore, the QNN-based model achieves faster processing times, with training and validation requiring 10.61 milliseconds per image and testing requiring 1.79 milliseconds per image.

A comparative performance analysis between the CRP_CNN_-ViT and CRP_QNN_-ViT models to evaluate the impact of incorporating Quanvolutional operations on classification performance is presented in Table 9. The CRP_QNN_-ViT model consistently outperformed the CRP_CNN_-ViT baseline, demonstrating improved training accuracy (98.18% vs. 97.44%) and validation accuracy (97.73% vs. 96.59%), with respective gains of 0.74% and 1.14%. The results clearly demonstrate that the CRP_QNN_-ViT model achieved a superior performance when compared to the CRP_CNN_-ViT model across all key metrics. To further investigate the performance variability of the models, a comparison was carried out using mean ± standard deviation values across five folds for both CRP_CNN_-ViT and CRP_QNN_-ViT architectures, as tabulated in Table 10. The CRP_QNN_-ViT model demonstrated a notable improvement in overall classification accuracy, achieving 97.73% ± 0.31, compared to 96.60% ± 0.03 for the CRP_CNN_-ViT model, reflecting an enhancement of +1.13%. While specificity and precision exhibited slight reductions of 0.08% and 0.10%, respectively, these differences fall within acceptable variance limits and do not negatively impact the model’s reliability. These findings highlight that the integration of quantum-inspired Quanvolutional layers into the CRP_QNN_-ViT architecture contributes to improved feature learning and generalization, thereby enhancing the model’s effectiveness in the classification of CRP.

### 4.2. Phase 2: Multi-Classification

The computational complexity and processing time for the CRP_CNN_-ViT and CRP_QNN_-ViT models are compared in Table 11. CRP_QNN_-ViT, which integrates quantum components, demonstrates notable improvements in efficiency over its classical counterpart. Specifically, CRP_QNN_-ViT requires only 86 million parameters compared to 132 million, as required by CRP_CNN_-ViT. Correspondingly, it achieves reductions in MACs and FLOPs, with values of 8.5 Giga MACs and 17 Giga FLOPs, respectively. In terms of processing speed, CRP_QNN_-ViT also offers faster inference and training times, requiring only 11.36 milliseconds for training and validation per image and 2.11 milliseconds for testing per image.

A detailed class-wise performance comparison carried out between the CRP_CNN_-ViT and CRP_QNN_-ViT models to assess the influence of Quanvolutional operations on multi-class classification outcomes is presented in Table 12. The CRP_QNN_-ViT model consistently exhibited improved or comparable performance across most evaluation metrics and class categories when compared to the CRP_CNN_-ViT, with the exception of a reduction in specificity and NPV for class 3. Importantly, the overall training and validation accuracy of the CRP_QNN_-ViT model reached 98.13% and 97.92%, respectively, outperforming CRP_CNN_-ViT, whose values were 97.28% and 96.02%, respectively. The comparison between both the DL models considered in this research as tabulated in Table 13 was taken into account for the 5-fold cross-validation. For most of the classes, the gains were more pronounced, though a marginal reduction was noted in NPV for class 2 and class 3.

While CNNs are highly optimized on classical hardware using GPUs and TPUs, they often face several limitations. CNNs typically exhibit high FLOP counts due to repeated convolutional and pooling operations, and deep architectures can contain millions of parameters, making them prone to overfitting and requiring extensive regularization. Additionally, memory usage in CNNs scales with model depth and input data size, leading to substantial resource demands.

QNNs, by contrast, leverage intrinsic properties of quantum computing such as superposition, entanglement, and quantum parallelism. These principles allow QNNs to perform multiple computations simultaneously, enabling more compact and efficient circuit representations. Quantum gates perform unitary transformations analogous to classical operations, but often with fewer operations when optimized. As a result, QNNs can operate with significantly fewer trainable parameters and reduced memory usage for model representation, as quantum states are encoded in the amplitudes of qubits.

Moreover, QNNs can represent complex functions in high-dimensional Hilbert spaces using fewer layers and less redundancy than classical deep networks, enhancing their theoretical representational power and learning capacity. However, simulating QNNs on classical machines remains computationally intensive as compared to quantum systems due to the exponential scaling of qubit states. This leads to high memory and processing demands, and the latency of simulated quantum gates does not match the efficiency expected from actual quantum hardware. Once mature, physical quantum systems are anticipated to alleviate these limitations through qubit-efficient computation and reduced memory footprints.

### 4.3. Challenges and Limitations

While the proposed CRP-ViT model has demonstrated strong performance in CRP classification, several challenges and limitations emerged during the course of this research. One of the primary concerns is data limitation. Additionally, the computational complexity of CRP_CNN_-ViT imposes significant demands on processing power and memory, which may restrict their deployment in real-time or resource-limited clinical settings. In general, high-resolution images are typically not used as direct input for either CNNs or QNNs for several reasons related to computational efficiency, memory limitations, and overfitting risk that diminishes the performance of the model. CRP_QNN_-ViT is constrained by the limited number of qubits available on near-term quantum devices, which restricts the model’s capacity to process high-dimensional medical imaging data directly. Scaling a QNN beyond four qubits introduces several challenges, including exponential growth in the Hilbert space, increased circuit depth, and susceptibility to noise and decoherence on the current hardware. As quantum hardware evolves to support more qubits with lower noise levels and improved gate fidelity, the CRP_QNN_-ViT framework can be systematically scaled by modular circuit expansion, qubit-efficient design patterns, and layer-wise quantum training techniques, thereby making it suitable for complex, high-dimensional medical imaging tasks.

Even though the performance of CRP_QNN_-ViT is superior, it imposes a barrier involving quantum hardware constraints, since current quantum processors are still in the early stages of development, with limited qubit fidelity and scalability. Future extensions of the quantum work will explore error mitigation techniques such as Zero-Noise Extrapolation (ZNE), and measurement error correction will be integrated to enhance inference fidelity. In the architecture, qubit-efficient encodings and hardware-efficient ansatzes will be incorporated to align circuit structures with native gate sets and the connectivity constraints of specific NISQ backends. These enhancements are expected to make CRP_QNN_-ViT more resilient to noise and thus more viable for deployment on near-term quantum hardware. Additionally, in future work, we intend to explore and systematically evaluate alternative entanglement strategies such as CZ, iSWAP, or hardware-efficient entanglement patterns through empirical experimentation and comparative analysis to better understand their impact on classification performance and circuit expressiveness.

## 5. Conclusions

This study presents a novel hybrid architecture, CRP_QNN_-ViT, which integrates quantum neural networks (QNNs) into a convolutional residual pathway-enhanced Vision Transformer framework for efficient CRP classification. The proposed model outperforms its classical counterpart, CRP_CNN_-ViT, in terms of both classification accuracy and computational efficiency across binary and multi-class tasks. Interestingly, the QNN model, outperforming the classical models, showed competitive results, particularly in generalizing to unseen data. This may be attributed to the ability of quantum circuits to represent complex data distributions with fewer parameters. However, the limitations of current quantum simulators, noise in quantum gates, and the lack of optimized quantum hardware may have impacted the full potential of the QNN model. Nonetheless, the results highlight that QNNs, when mature, could offer a powerful alternative for high-dimensional medical imaging tasks, especially where classical models face scalability issues.

## Figures and Tables

**Table 1:** Summary of the proposed CRP_QNN_-ViT architecture.

Stage	Layer Type	Details/Description	No. of Layers	Output Size
Input	Quantum-Encoded Input Image	256 × 256 × 3	–	256 × 256 × 3
Stage 0	Quantum Conv + Quantum Pooling	7 × 7 QConv, 64 filters, stride 2 + 3 × 3 QPool	2	56 × 56 × 64
Stage 1	Quantum Conv Block + 2 Identity QBlocks	[1 × 1, 64] → [3 × 3, 64] → [1 × 1, 256]	3 blocks (9 layers)	56 × 56 × 256
Stage 2	Quantum Conv Block + 3 Identity QBlocks	[1 × 1, 128] → [3 × 3, 128] → [1 × 1, 512]	4 blocks (12 layers)	28 × 28 × 512
Stage 3	Quantum Conv Block + 5 Identity QBlocks	[1 × 1, 256] → [3 × 3, 256] → [1 × 1, 1024]	6 blocks (18 layers)	14 × 14 × 1024
Stage 4	Quantum Conv Block + 2 Identity QBlocks	[1 × 1, 512] → [3 × 3, 512] → [1 × 1, 2048]	3 blocks (9 layers)	7 × 7 × 2048
Pooling	Quantum Global Average Pooling	Reduces feature map to 1 × 1 × 2048	1	1 × 1 × 2048
FC Layer	Quantum Fully Connected + QSigmoid/QSoftmax	Dense layer with QSigmoid/QSoftmax (intermediate feature vector)	1	2048 or custom size
Patch Embedding	Patchify + Linear Projection	Convert 2D features to patch tokens (e.g., 16 × 16 patches)	1	N × D (e.g., 196 × 768)
Transformer Encoder	Multi-Head Self-Attention + MLP	Multiple transformer blocks with LayerNorm and MLP	12 blocks typical	N × D (e.g., 196 × 768)
Classification Head	MLP Head + Sigmoid for Binary/ MLP Head + Softmax for Multi-Class	Final classification from [CLS] token	1	2 for binary/ 4 for multi-class

**Table 2:** Performance metrics achieved by CRP_QNN_-ViT (binary class) using 80:20 split.

Task	Epoch	Accuracy	Sensitivity	Specificity	Precision	NPV
Training	23	98.18	98.41	97.95	97.96	98.4
Validation		97.73	98.64	96.82	96.88	98.61

Note: All performance metrics are expressed in percentage format.

**Table 3:** Performance metrics achieved by CRP_QNN_-ViT (binary class) using 5-fold cross-validation.

K-Fold	Accuracy	Sensitivity	Specificity	Precision	NPV
K = 1	98.18	99.09	97.27	97.27	99.07
K = 2	97.95	99.09	96.82	96.82	99.07
K = 3	97.27	98.18	96.36	96.36	98.15
K = 4	97.5	98.64	96.36	96.36	98.6
K = 5	97.73	98.64	96.82	96.82	98.61
Mean ± SD	97.73 ± 0.31	98.73 ± 0.38	96.73 ± 0.33	96.73 ± 0.33	98.70 ± 0.36

Note: All performance metrics are expressed in percentage format.

**Table 4:** Difference between 80:20 split and 5-fold cross-validation of CRP_QNN_-ViT (binary class).

K-Fold	Accuracy	Sensitivity	Specificity	Precision	NPV
80:20 Split (A)	97.73	98.64	96.82	96.88	98.61
5-Fold (B)	97.73	98.73	96.73	96.73	98.7
Difference Between (A and B)	00.00	0.09	0.09	0.15	0.09

Note: All performance metrics are expressed in percentage format.

**Table 5:** Performance metrics achieved by CRP_QNN_-ViT (multi-class) using 80:20 split.

Optimizers	Epoch	Class	Accuracy	Sensitivity	Specificity	Precision	NPV	Overall Accuracy
Training	29/50	0	99.15	98.59	99.72	99.15	99.53	98.13
		1	98.48	97.38	99.49	98.48	99.11	
		2	97.82	97.73	99.27	97.82	99.24	
		3	97.06	98.84	99.03	97.06	99.62	
Validation		0	98.86	98.49	99.62	98.87	98.86	97.92
		1	98.11	97.37	98.49	98.11	98.11	
		2	97.73	97.36	98.11	97.73	97.73	
		3	96.97	98.46	95.09	96.97	96.97	

Note: All performance metrics are expressed in percentage format.

**Table 6:** Performance metrics achieved by CRP_QNN_-ViT (multi-class) using 5-fold cross-validation.

K-Fold	Class	Accuracy	Sensitivity	Specificity	Precision	NPV	Overall Accuracy
K = 1	0	98.48	98.48	99.15	98.48	99.15	97.54
	1	96.64	96.64	99.05	96.64	99.05	
	2	96.98	96.98	98.86	97.36	98.84	
	3	98.07	98.07	97.59	96.21	98.86	
K = 2	0	98.5	98.5	99.62	99.62	98.5	98.39
	1	97.75	97.75	98.86	98.49	98.12	
	2	98.11	98.11	98.11	98.11	98.11	
	3	99.22	99.22	98.42	96.97	99.36	
K = 3	0	98.49	98.49	98.86	98.49	98.86	97.82
	1	97.74	97.74	98.11	98.11	97.74	
	2	96.98	96.98	97.35	97.35	96.98	
	3	98.08	98.08	96.97	96.97	98.08	
K = 4	0	98.5	98.5	99.24	99.24	98.5	98.2
	1	98.11	98.11	98.48	98.48	98.11	
	2	97.73	97.73	97.73	97.73	97.73	
	3	98.47	98.47	97.35	97.35	98.47	
K = 5	0	98.12	98.12	98.86	98.86	98.12	97.73
	1	97.36	97.36	97.73	97.73	97.36	
	2	97.35	97.35	97.35	97.35	97.35	
	3	98.08	98.08	96.97	96.97	98.08	
Mean ± SD	0	98.42 ± 0.17	98.42 ± 0.17	99.15 ± 0.32	98.94 ± 0.49	98.63 ± 0.39	97.94 ± 0.35
	1	97.52 ± 0.56	97.52 ± 0.56	98.45 ± 0.54	97.89 ± 0.77	98.08 ± 0.63	
	2	97.43 ± 0.49	97.43 ± 0.49	97.88 ± 0.63	97.58 ± 0.34	97.80 ± 0.72	
	3	98.38 ± 0.50	98.38 ± 0.50	97.46 ± 0.60	96.89 ± 0.42	98.57 ± 0.55	

Note: All performance metrics are expressed in percentage format.

**Table 7:** Difference between 80:20 split and 5-fold cross-validation of CRP_QNN_-ViT (multi-class).

Method	Class	Accuracy	Sensitivity	Specificity	Precision	NPV	Overall Accuracy
80:20 Split (A)	0	98.86	98.49	99.62	98.87	98.86	97.92
	1	98.11	97.74	98.49	98.11	98.11	
	2	97.73	97.36	98.11	97.73	97.73	
	3	96.97	98.08	95.85	96.97	96.97	
5-Fold (B)	0	98.42	98.42	99.15	98.94	98.63	97.94
	1	97.52	97.52	98.45	97.89	98.08	
	2	97.43	97.43	97.88	97.58	97.80	
	3	98.38	98.38	97.46	96.89	98.57	
Difference Between (A and B)	0	0.44	0.07	0.47	−0.07	0.23	−0.02
	1	0.59	0.22	0.04	0.22	0.03	
	2	0.3	−0.07	0.23	0.15	−0.07	
	3	−1.41	−0.30	−1.61	0.08	−1.60	

Note: All performance metrics are expressed in percentage format.

**Table 8:** Comparison of computational parameters of CRP_CNN_-ViT and CRP_QNN_-ViT (binary class).

Method	Model	Computational Complexity/Load			Time	
		Total Parameters (Million)	MACs (Giga)	FLOPs (Giga)	Training and Validation Per Image (Milliseconds)	Testing Per Image (Milliseconds)
CRP_CNN_-ViT [56]	CNN	132	11.6	23.2	17.69	3.58
CRP_QNN_-ViT	QNN	86	8.5	17	10.61	1.79

Note: All performance metrics are expressed in percentage format.

**Table 9:** Comparison of key metrics of CRP_CNN_-ViT and CRP_QNN_-ViT (binary class) using 80:20 split.

Method	Epoch	Training					Validation				
		Accuracy	Sensitivity	Specificity	Precision	NPV	Accuracy	Sensitivity	Specificity	Precision	NPV
CRP_CNN_-ViT [56]	20	97.44	97.61	97.28	97.27	97.61	96.59	96.38	96.80	96.82	96.36
CRP_QNN_-ViT	23	98.18	98.41	97.95	97.96	98.4	97.73	98.64	96.82	96.88	98.61
Difference	3	0.74	0.8	0.67	0.69	0.79	1.14	2.26	0.02	0.06	2.25

Note: All performance metrics are expressed in percentage format.

**Table 10:** Comparison of key metrics of CRP_CNN_-ViT and CRP_QNN_-ViT (binary class) using 5-fold cross-validation.

Method	Accuracy	Sensitivity	Specificity	Precision	NPV
CRP_CNN_-ViT (A) [56]	96.60 ± 0.03	96.41 ± 0.03	96.81 ± 0.02	96.83 ± 0.02	96.37 ± 0.02
CRP_QNN_-ViT (B)	97.73 ± 0.31	98.73 ± 0.38	96.73 ± 0.33	96.73 ± 0.33	98.70 ± 0.36
Difference Between (A and B)	+1.13 ± 0.31	+2.32 ± 0.38	−0.08 ± 0.33	−0.10 ± 0.33	+2.33 ± 0.36

Note: All performance metrics are expressed in percentage format.

**Table 11:** Comparison of computational parameters of CRP_CNN_-ViT and CRP_QNN_-ViT (multi-class).

Method	Model	Computational Complexity/Load			Time	
		Total Parameters (Million)	MACs (Giga)	FLOPs (Giga)	Training and Validation Per Image (Milliseconds)	Testing Per Image (Milliseconds)
CRP_CNN_-ViT [57]	CNN	132	11.6	23.2	18.93	4.20
CRP_QNN_-ViT	QNN	86	8.5	17	11.36	2.11

Note: All performance metrics are expressed in percentage format.

**Table 12:** Comparison of key metrics of CRP_CNN_-ViT and CRP_QNN_-ViT (multi-class) using 80:20 split.

Model	Epoch	Class	Training						Validation					
			Accuracy	Sensitivity	Specificity	Precision	NPV	Overall Accuracy	Accuracy	Sensitivity	Specificity	Precision	NPV	Overall Accuracy
CRP_CNN_-ViT [57]	22/50	0	97.92	97.55	99.31	97.44	98.9	97.28	98.01	97.75	98.29	98.86	98.51	96.02
		1	96.44	96.44	99.13	97.25	98.5		95.93	93.8	97.97	97.35	96.2	
		2	97.16	97.16	99.15	96.5	98.75		96.57	95.08	96.68	95.08	97.04	
		3	97.98	97.98	99.43	97.98	99		96.97	97.61	96.8	92.8	99.02	
CRP_QNN_ -ViT	29/50	0	99.15	98.59	99.72	99.15	99.53	98.13	98.86	98.49	99.62	98.87	98.86	97.92
		1	98.48	97.38	99.49	98.48	99.11		98.11	97.37	98.49	98.11	98.11	
		2	97.82	97.73	99.27	97.82	99.24		97.73	97.36	98.11	97.73	97.73	
		3	97.06	98.84	99.03	97.06	99.62		96.97	98.46	95.09	96.97	96.97	
Difference	7	0	1.23	1.04	0.41	1.71	0.63	0.85	0.85	0.74	1.33	−0.01	0.35	1.9
		1	2.04	0.94	0.36	1.23	0.61		2.18	3.57	0.52	0.76	1.91	
		2	0.66	0.57	0.12	1.32	0.49		1.16	2.28	1.43	2.65	0.69	
		3	−0.92	0.86	−0.40	−0.92	0.62		0	0.85	−1.71	4.17	−2.05	

Note: All performance metrics are expressed in percentage format.

**Table 13:** Comparison of key metrics of CRP_CNN_-ViT and CRP_QNN_-ViT (multi-class) using 5-fold cross-validation.

Method	Class	Accuracy	Sensitivity	Specificity	Precision	NPV	Overall Accuracy
CRP_CNN_-ViT (A) [57]	0	98.22 ± 0.94	96.87 ± 0.86	98.64 ± 0.85	97.88 ± 1.24	98.19 ± 0.95	96.27 ± 1.06
	1	97.07 ± 1.02	95.24 ± 1.22	97.70 ± 1.21	96.29 ± 1.65	97.42 ± 0.81	
	2	96.77 ± 1.06	95.61 ± 1.04	97.15 ± 1.33	95.00 ± 1.61	97.94 ± 0.98	
	3	96.89 ± 1.55	97.42 ± 1.45	96.43 ± 1.68	94.09 ± 1.19	98.99 ± 0.69	
CRP_QNN_-ViT (B)	0	98.42 ± 0.17	98.42 ± 0.17	99.15 ± 0.32	98.94 ± 0.49	98.63 ± 0.39	97.94 ± 0.35
	1	97.52 ± 0.56	97.52 ± 0.56	98.45 ± 0.54	97.89 ± 0.77	98.08 ± 0.63	
	2	97.43 ± 0.49	97.43 ± 0.49	97.88 ± 0.63	97.58 ± 0.34	97.80 ± 0.72	
	3	98.38 ± 0.50	98.38 ± 0.50	97.46 ± 0.60	96.89 ± 0.42	98.57 ± 0.55	
Difference Between (A and B)	0	+0.20 ± 0.96	+1.55 ± 0.88	+0.51 ± 0.91	+1.06 ± 1.34	+0.44 ± 1.03	+1.67 ± 1.11
	1	+0.45 ± 1.16	+2.28 ± 1.35	+0.75 ± 1.33	+1.60 ± 1.83	+0.66 ± 1.03	
	2	+0.66 ± 1.17	+1.82 ± 1.14	+0.73 ± 1.47	+2.58 ± 1.65	−0.14 ± 1.23	
	3	+1.49 ± 1.63	+0.96 ± 1.55	+1.03 ± 1.78	+2.80 ± 1.26	−0.42 ± 0.88	

Note: All performance metrics are expressed in percentage format.

## Data Availability

The data that support the findings of this study are openly available at the following. (1) URL: https://figshare.com/articles/figure/Polyp_DataSet_zip/21221579?file=37636550 (accessed on 16 July 2025); DOI: https://doi.org/10.1016/j.patcog.2012.03.002. (2) URL: https://www.kaggle.com/datasets/balraj98/cvcclinicdb (accessed on 16 July 2025); DOI: https://doi.org/10.1016/j.compmedimag.2015.02.007. (3) URL: http://www.depeca.uah.es/colonoscopy_dataset/ (accessed on 16 July 2025); DOI: https://doi.org/10.1109/tmi.2016.2547947.
